# Interaction between age and fatigue on antagonist muscle coactivation during an acute post-fatigue recovery phase

**DOI:** 10.3389/fragi.2022.1005080

**Published:** 2022-10-03

**Authors:** Sara A. Harper, Brennan J. Thompson

**Affiliations:** ^1^ Department of Kinesiology and Health Science, Utah State University, Logan, UT, United States; ^2^ Sorenson Legacy Foundation Center for Clinical Excellence, Dennis Dolny Movement Research Clinic, Utah State University, Logan, UT, United States

**Keywords:** aging, electromyography, biceps femoris, older adults, quadriceps, hamstrings, isometric contraction

## Abstract

This study investigated the age-related changes in antagonist muscle coactivation of the biceps femoris (BF) during an acute recovery period following a leg extensor fatiguing protocol. Twenty-three young (mean ± SD: age = 25.1 ± 3.0 years) and twenty-three old men (age = 71.5 ± 3.9 years) participated. Surface electromyography (sEMG) was recorded from the BF muscles for antagonist muscle coactivation. Testing involved participants performing leg extension isometric maximal voluntary contractions (MVCs) and isokinetic MVCs at 240°·s^−1^ at baseline (Pre) and again after the fatigue protocol at 0 (Post0), 7 (Post7), 15 (Post15), and 30 (Post30) minutes post fatigue. Root mean square (RMS) values were computed from the BF sEMG and were calculated as the first 200 ms from onset for the isometric (IsomCoact200ms) and dynamic isokinetic 240°·s^−1^ (DynCoact200ms) MVCs, and for the final 10° of the leg extension (DynCoact10°) on the isokinetic 240°·s^−1^ MVCs. Two-way ANOVAs [age group (young vs. old) × time (Pre vs. Post0 vs. Post7 vs. Post15 vs. Post30)] showed that DynCoact200ms had an effect for time (*p* = 0.018), with greater antagonist coactivation in Pre than Post0 (*p* = 0.009) and recovering by Post7 (*p* = 0.011) with no group differences. DynCoact10° had no age × time interaction (*p* = 0.070), but had a main effect for time (*p* = 0.020) with the Post0 being lower than the Pre. However, for this variable the young group showed a more severe Pre to Post0 fatigue decline (−45.9%) than the old group (−6.7%) indicating this may be a more sensitive variable for capturing age-related antagonist coactivation post-fatigue responses. Leg extensor fatigue affects some BF coactivation sEMG variables more than others, and any altered post-fatigue coactivation response recovers rapidly (<7 min) from baseline levels.

## 1 Introduction

Fatigue is a prominent risk factor for reduced function and independence and increased falls in older adults (Nagano et al., 2014; Morrison et al., 2016; Kamitani et al., 2019; Renner et al., 2021). The effects of fatigue on older adult muscle function have been rather extensively studied (Katsiaras et al., 2005; Kent-Braun, 2009; Seene and Kaasik, 2012; Foulis et al., 2017). Interestingly, older adults often exhibit reduced relative fatigue effects compared to younger adults as a result of fatiguing contractions (Allman and Rice, 2002; Kent-Braun, 2009; Avin and Frey Law, 2011), or overall better performance on time to task failure at the same relative, submaximal fatiguing load (Hunter et al., 2005; Enoka and Duchateau, 2008; Griffith et al., 2010; Avin and Frey Law, 2011). Effective management of fatigue in older persons to enhance quality of life and functional living involves a two-pronged approach, namely, minimizing fatigue and enhancing recovery to full function when fatigue has occurred.

The effect of fatigue when comparing young and old subjects has been shown to be dependent on the type and mode of exercise used (prolonged continuous vs. intermittent, isometric vs. dynamic, fast vs. slow contraction speed, isokinetic vs. isotonic contractions) (Lanza et al., 2004; Dalton et al., 2010; Dalton et al., 2012; Senefeld et al., 2017; Wu et al., 2019). Although a modest level of fatigue resistance may be present in older muscle under specific conditions (Katsiaras et al., 2005; Chung et al., 2007; Kent-Braun, 2009; Avin and Frey Law, 2011), less research has been conducted in regards to recovery patterns after fatigue occurrence among older adults (Conchola et al., 2013; Foulis et al., 2017).

As changes occur at the neuromuscular level with increased age, age-related fatigue resistance characteristics could provide insight on acute recovery and physical function implications. For example, prior work has shown that muscle activation as assessed *via* surface electromyography (sEMG) may change with fatigue (Kent-Braun, 2009; Foulis et al., 2017), and that a combination of motor unit behavior and muscle contractile properties may contribute to resistance to muscle fatigue in healthy older adults compared to younger adults (Kent-Braun, 2009). Resisting muscle fatigue may have important implications for physical function (Kent-Braun, 2009; Foulis et al., 2017), as for instance, age-related reductions in physical function are influenced by decreased mechanical power (Bassey et al., 1992; Reid and Fielding, 2012) which is a common outcome resulting from fatigue. Therefore, understanding specific neuromuscular changes induced by fatigue in older adults may provide critical insight on fatigue resistance characteristics which could potentially help preserve physical function (Kent-Braun, 2009).

Muscle coactivation, typically defined as the unintentional concurrent activation of the antagonist muscles at a given joint with agonist muscle activation (Latash, 2018), may increase in old age (Hortobágyi and DeVita, 2006; Arellano et al., 2016). For example, old compared to young adults have been shown to exhibit twice the coactivation amplitude for the wrist extensors (Arellano et al., 2016). In regard to fatigue, older adults appear to have enhanced coactivation during down stepping (Hortobágyi and DeVita, 2000), and during walking with balance perturbations (Thompson et al., 2018). While during an activity, increasing joint stabilization may be considered as a positive attribute in some scenarios, in acute recovery Power et al. (2010) found a 13 ± 9% increase in ankle dorsiflexor’s coactivation for up to 20 min, before returning to baseline by 30 min. This enhanced coactivation could be considered a negative attribute, increasing stiffness and fall risk (Hortobágyi et al., 2009; Thompson et al., 2018). Of considerable consequence is that increased coactivation after fatigue may be a contributing factor for reduced muscle power (Foulis et al., 2017), which may be a risk factor for worsened physical function, injury risk, and postural control in older adults.

The role of neuromuscular fatigue on coactivation and the subsequent recovery response patterns requires further investigation. In particular, Power et al. (2010) reported coactivation in regard to acute recovery patterns after a fatigue-inducing bout for young adults, but research is lacking on how the effects may diverge in young compared to older adults in recovery. Specifically, the antagonist leg flexors arguably serve its most relevant protective function at the knee joint during knee extension movements. Namely, the leg flexors function to stabilize and protect the knee joint while actively contributing to limb deceleration through range of motion (ROM) (Baratta et al., 1988; Aagaard et al., 2000). Therefore, the purpose of this investigation was to examine the acute antagonist muscle coactivation recovery responses following a fatigue-inducing bout involving the leg extensors in young and older adults focusing on coactivity from the biceps femoris (BF) muscles. We hypothesized that the old group would have enhanced antagonist muscle coactivation after fatigue and into recovery, with no changes in the young group in the 30 min period following the fatigue bout.

## 2 Materials and methods

### 2.1 Participants

Twenty-three young men (mean ± SD: age = 25.1 ± 3.0 years; height = 178.8 ± 7.7 cm; mass = 88.1 ± 21.5 kg) and twenty-three old men (age = 71.5 ± 3.9 years; height = 177.9 ± 5.9 cm; mass = 88.7 ± 12.6 kg) volunteered to participate in the study. All participants were apparently healthy and recreationally active. Eligibility criteria for the study were that they must have a body mass index of between 18 and 40 kg/m^2^, have no lower limb injuries nor neuromuscular disorders (e.g., Parkinson’s disease), could not be involved in elite level sports (collegiate, semi-professional, or masters athletics), could not be involved in high level training routines (training for marathon, triathlon or competitive weightlifting, etc.), and were deemed to be otherwise healthy with no serious health disorders that would preclude them from performing strenuous physical activity. The average self-reported level of structured physical activity (aerobic, strength training, and sport participation combined) per week was 1.3 h for the young and 2.4 h for the old groups. The present study uses a subset of the data obtained from a larger investigation (Conchola et al., 2013; Thompson et al., 2015), however, the present data and research question have not been a part of any other investigation. The study procedures were approved by a University Institutional Review Board and participants read and signed an informed consent document prior to participation.

### 2.2 Procedures

The procedures have been described in detail previously (Conchola et al., 2013; Thompson et al., 2015). Briefly, participants visited the laboratory on two occasions separated by 48–96 h. The first visit was used to familiarize participants with both maximal voluntary contractions (MVCs) and the fatigue protocol. Participants were instructed to refrain from vigorous physical activity 48 h prior to the session and avoid caffeine consumption 12 h before the testing. On the second visit, participants performed baseline MVCs, followed by the fatigue protocol, and then follow-up MVCs.

#### 2.2.1 Surface electromyography

Prior to testing, participants performed a 5 min warm-up on a cycle ergometer at a self-selected, low intensity workload and then were prepped and fitted with the sEMG electrodes. The sEMG was recorded from the BF muscle, which served as the antagonist for the leg extension MVC assessments. Following skin preparation, pre-gelled bipolar surface electrodes (EL502, Biopac Systems Inc., Goleta, CA, United States; inter-electrode distance = 25 mm) were placed over the skin at 50% of the distance between the ischial tuberosity and lateral tibial condyle of the right, posterior thigh (e.g., BF) and a reference electrode was placed on the tibial tuberosity.

#### 2.2.2 Dynamometer testing

All MVCs were performed with the right limb on a Biodex System 4 dynamometer (Biodex Medical Systems, Inc. Shirley, NY, United States) in accordance with our previously reported procedures (Thompson et al., 2015). A specific warm-up was performed where submaximal leg extensions were performed at 60°·s^−1^ at 75% of their perceived maximal effort. Participants then performed two isometric leg extension MVCs at a leg angle of 60°, followed by two concentric isokinetic MVCs at 240°·s^−1^ through an 80° range of motion. The same testing pattern was performed immediately following the fatigue protocol considered as time 0 (Post0), and again at 7 (Post7), 15 (Post15), and 30 (Post30) minutes into recovery. During the familiarization session only, two isometric leg flexion MVCs were performed as a means to provide maximal leg flexor sEMG activation for normalization of the BF sEMG variables.

#### 2.2.3 Fatigue protocol

The highest baseline isometric MVC peak torque value was used for calculating the target load for the fatigue protocol which was set at 60% of the isometric MVC. Five minutes following the baseline MVCs, participants performed the fatigue protocol which included intermittent isometric contractions using a 0.6 duty cycle (i.e., 6 s contraction and 4 s relaxation phase) (Bigland-Ritchie and Woods, 1984; Vollestad et al., 1997; Thompson et al., 2015). For the intermittent contractions, participants viewed their real-time torque output which was plotted using a target torque line on a computer placed directly in front of them. When participants were no longer able to reach their target torque level, the test was terminated. Endurance time (i.e., time until exhaustion) was the outcome measure used to assess fatigue performance.

#### 2.2.4 Signal processing

The sEMG signals were sampled at 2 kHz with a Biopac data acquisition system (MP150WSW, Biopac Systems, Inc.; Santa Barbara, CA, United States) and processed offline with custom written software (LabVIEW 2018, National Instruments, Austin, TX, United States). The sEMG signals (µV) were band pass filtered at 20–400 Hz using a zero phase shift, fourth-order Butterworth filter (Conchola et al., 2013). The following outcome measures were calculated as the normalized root mean square (RMS) value from the sEMG signals: 1) isometric BF coactivation RMS at 0–200 ms (IsomCoact200ms), 2) dynamic isokinetic 240°·s^−1^ BF coactivation RMS at 0–200 ms (DynCoact200ms), and 3) BF coactivation for the final 10° of the leg extension ROM (DynCoact10°) for the isokinetic 240°·s^−1^ MVCs. Note, an approximate limb range of motion covered during the isokinetic contraction from the first 200 ms from EMG onset for a subsample of the subjects was between 3 and 11°. All sEMG signals were expressed as normalized values relative to the RMS value corresponding to the point (500 ms epoch) where peak torque occurred for the isometric leg flexion MVCs (i.e., the resulting values are the antagonist BF sEMG activity during the leg extension movement expressed as the % of BF sEMG activation to its own agonist isometric MVC sEMG value; the resulting values are thus expressed in % units). For the IsomCoact200ms and DynCoact200ms variables, the RMS was calculated during the first 200 ms of the contraction from sEMG onset which was manually determined as described previously (Conchola et al., 2013). The RMS during the first 200 ms of the contraction represents the early or rapid activation characteristics of the muscle which early time point may be differentially affected by fatigue compared to the final ROM activation characteristics (i.e., DynCoact10°). The DynCoact10° RMS was calculated for the final 10° of the leg extension ROM.

#### 2.2.5 Data analysis

Two-way mixed factorial analyses of variance (ANOVAs) [age group (young vs. old) × time (Pre vs. Post0 vs. Post7 vs. Post15 vs. Post30)] were performed to evaluate the effects of fatigue and age group on antagonist muscle coactivation variables immediately after fatigue and throughout a 30 min recovery period. Interaction values and/or the main effect of time was displayed when appropriate. Follow-up analyses included one-way repeated measures ANOVAs and Bonferroni-corrected pairwise comparisons. A univariate scatterplot which depicts individual subject data was created using templates from Weissgerber et al. (2015). All data was analyzed using the Statistical Package for Social Sciences software (IBM Statistics v.25, Chicago, IL, United States). Alpha was set *a priori* at *p* ≤ 0.05. GraphPad Software was used to visually depict data (GraphPad Software, San Diego, CA, United States).

## 3 Results

**FIGURE 1:**
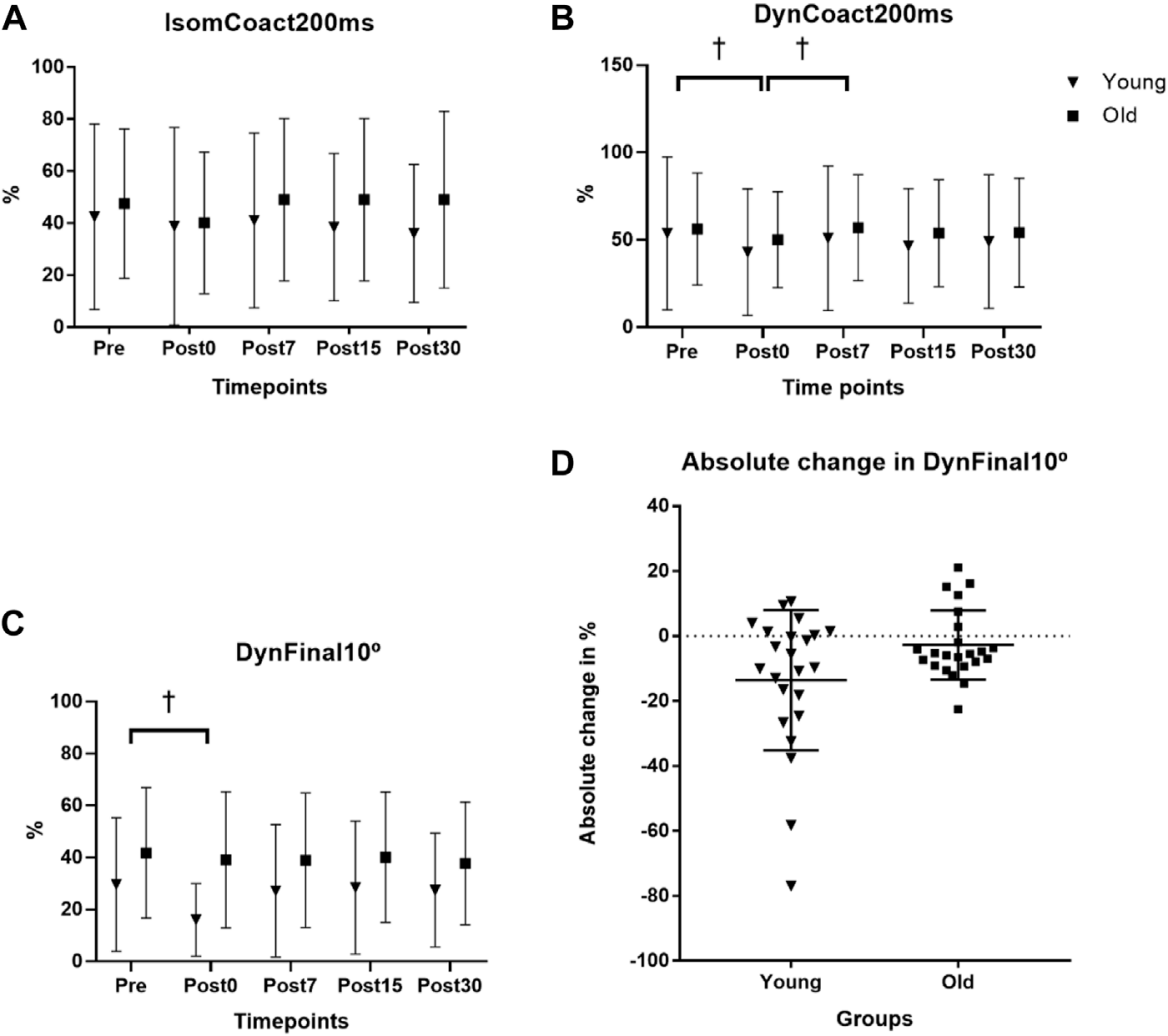
Effects of age and fatigue on antagonist muscle coactivation. **(A)** The isometric biceps femoris (BF) antagonist muscle coactivation RMS at 0–200 ms (IsomCoact200ms) variable had no interaction (*p* = 0.237) nor main effect for time (*p* = 0.277). **(B)** The dynamic isokinetic 240°·s^−1^ BF coactivation RMS at 0–200 ms (DynCoact200ms) variable had no interaction (*p* = 0.802), but there was a main effect for time (*p* = 0.018). Follow up analyses with collapsed groups showed that Pre was higher than Post0 (*p* = 0.009), and Post7 was higher than Post0 (*p* = 0.011, depicted by †). **(C)** BF coactivation for the final 10° of the leg extension range of motion (DynCoact10°) for the isokinetic 240°·s^−1^ MVCs showed no interaction (*p* = 0.070), but there was a main effect for time (*p* = 0.020). Follow up analyses with collapsed groups showed that Pre was higher than Post0 (*p* = 0.022, depicted by †). Data for **(A–C)**, represent mean ± SD. **(D)** A univariate scatterplot depicts the absolute change in sEMG RMS for the DynCoact10° variable from before (Pre) to immediately after (Post0) the fatigue protocol for the young and old groups. Horizontal bars represent the group means (young = −13.59% ± 21.54%; old = −2.72% ± 10.63%).

The endurance time fatigue outcome has been reported and discussed previously (Thompson et al., 2015). Briefly, the fatigue protocol elicited a 38.0% and 38.4% reduction in isometric strength at the posttest in the young and old men, respectively, with the old men showing a non-significantly longer (443.3 ± 364.4 s; *p* = 0.085) endurance time compared to the young men (274.3 ± 191.8 s). For IsomCoact200ms, there was no age × time interaction (*p* = 0.237) nor main effect for time (*p* = 0.277; see Figure 1A). For DynCoact200ms, there was no age × time interaction (*p* = 0.802), but there was a main effect for time (*p* = 0.018). Follow up analyses with collapsed groups showed that BF coactivation was lower for Post0 compared to both Pre (*p* = 0.009) and Post7 (*p* = 0.011) time points with no other time points reaching statistical significance (Figure 1B). For DynCoact10°, there was no age × time interaction (*p* = 0.070), but there was a main effect for time (*p* = 0.020). Follow up analyses with collapsed groups showed that Pre was higher than Post0 (*p* = 0.022; see Figure 1C). A scatterplot shows the absolute change in sEMG RMS for the DynCoact10° variable from before (Pre) to immediately after (Post0) the fatigue protocol (see Figure 1D). Horizontal bars represent the group means (Young: −13.5 vs. Old: −2.7). Marginal means for the time factor (collapsed across groups) are reported for IsomCoact200ms, DynCoact200ms, and DynCoact10° in Supplementary Table S1.

## 4 Discussion

The purpose of this investigation was to examine the acute BF antagonist muscle coactivation responses within a 30 min period following a fatigue-inducing bout involving the leg extensors in young and old men. We hypothesized that old adults would have enhanced antagonist muscle coactivation following fatigue and extending into the recovery phase, whereas no changes would be observed in the young adults. Based on our results, we reject this hypothesis on the basis that the old group did not show an increase in coactivation for any variable and that a depressed coactivation response was shown immediately following fatigue for both groups for the DynCoact200ms, and the DynCoact10° variables.

The DynCoact200ms variable revealed a depressed (∼15% *via* marginal means) BF coactivation response immediately following the fatigue bout, with age group collapsed (Figure 1B). It appears that when assessing BF coactivation *via* the isometric MVC measure there is not any indication of change to the coactivation amplitude resulting from fatigue of the agonist (leg extensors) muscle group. The reason for this could be related to the static nature of the isometric task, which due to it being more inherently stable, may not be highly sensitive for inducing any physiological alterations to the antagonist’s coactivity level. It is notable that given the IsomCoact200ms and DynCoact200ms variables assessed the sEMG RMS values at the same time point of 200 ms from the sEMG signal onset, it appears that the dynamic nature of the 240°·s^−1^ may provide a better (i.e., *via* heightened sensitivity) assessment of the fatigue-related coactivation response since the BF coactivation would likely be more sensitive to its antagonistic functions during a rapid dynamic contraction. The finding that DynCoact200ms contraction decreased with fatigue is interesting and may represent a reduction in overall neural drive with the occurrence of fatigue, or perhaps a compensatory mechanism whereby the antagonist reduces activation to compensate for the loss of force/power producing capacities resulting from fatigue. Indeed, a common strategy to overcome muscular fatigue involves reducing antagonist activation, which may alter knee joint stability, and plausibly lead to increased injury risk (Padua et al., 2006).

These results revealed that for the DynCoact10° variable there was a main effect for time (*p* = 0.020) such that the collapsed groups showed that Post0 was depressed by 22.8% compared to Pre (*p* = 0.022; see Figure 1C). However, it should be noted that the interaction statistic for this variable approached statistical significance (*p* = 0.070) and a close inspection of the group data for this variable did seem to show somewhat of a divergence between groups (see Figure 1D scatterplot). In fact, the young group showed a −45.9% reduction from Pre to Post0, whereas the old group only had a 6.7% reduction. The nonsignificant interaction of *p* = 0.070 is likely a consequence of the high variance that is typically associated with EMG. Thus, the non-significant interaction should be interpreted with caution as it is possible there is actually an effect of age on the fatigue-induced antagonist (BF muscle) coactivation response for the final 10° ROM following fatigue of the leg extensors acting as agonist. Nevertheless, based on the statistical outcomes of this study the groups overall had reduced antagonist coactivation immediately following fatigue, which may have contributed to a depressed muscular power output proportionally more than young at this post fatigue time point. Such findings would align with Padua et al. (2006) that observed a -26% (F = 12.073, *p* = 0.002) reduction in hamstring antagonist coactivation in young adults after a fatiguing protocol. While the present study design is not suited to determine the mechanistic causes underpinning the findings, it may be speculated that the older men may have an impaired gamma feedback loop, related to age decreases in muscle spindle sensitivity as has been suggested in previous work (Konishi et al., 2007; Ryan et al., 2014). Such an impairment, if present in old adults, would theoretically reduce the sensitivity of the antagonist coactivation response resulting from neuromuscular fatigue. Since there was a more severe reduction in DynCoact10° BF coactivation at Post0 (compared to Pre) in young (−45.9%), vs. the old (−6.7%) adults, it would suggest the large reduction is the “healthy” neuromuscular response to the fatigue. The tempered response in old adults for this coactivation measure is a unique finding that warrants further investigation in regard to both mechanistic and functional implications. It should be noted that for the two variables where coactivation depression was observed at Post0 (DynCoact200ms and DynFinal10°), the values were not depressed, when compared to Pre, for any of the recovery time points (Post7, 15, 30). This suggests a rapid recovery of the depressed coactivation response that was induced immediately after the fatigue bout. In this context, it appears that a lengthy recovery period may not be required for restoration of the antagonist’s coactivation capacities.

The present study revealed a novel variable that is well-suited for representing the antagonist (i.e., BF as a representation of the hamstrings) muscle during dynamic leg extension coactivation. Namely, the DynCoact10° was arguably shown to be the most sensitive variable in capturing changes in the BF coactivation from fatigue likely due to assessing the antagonist activation specifically at the final 10° of the ROM. The final 10° would be an important point in the ROM as it is the point in the dynamic ROM that most likely reflects the antagonists most important role during the dynamic leg extension rapid movement by providing knee stabilization and leg deceleration functions. DynCoact10° may thus be warranted as a variable in future work examining dynamic coactivation tasks, and in particular, may be a useful measure to reflect the functional stabilization role of the BF coactivation during leg extension dynamic movements.

A notable limitation of our approach of assessing antagonist muscle coactivation during leg extension is the use of assessing the sEMG activation of the BF muscle (as a sole, more lateral measure of leg flexor’s coactivation), to represent the total antagonist leg flexor muscle group coactivation. It is possible that assessing the semimembranosus muscle, which would represent more medial hamstring coactivation, may influence the functional interpretation of the hamstring’s action as an antagonist muscle group (Sisante et al., 2020). In addition, we did not account for the amount of subcutaneous adipose tissue at the BF sEMG placement site which, due to volume-conducted cross talk, may affect the interpretation of our results between the young and old groups. Indeed, after Wu et al. (2017) controlled for subcutaneous adipose tissue, their older group had higher antagonist coactivation compared to a young group. However, the present study sample had no differences in body mass index between the two groups (young 27.6 kg/m^2^; old 28.0 kg/m^2^ body mass index), and so the potential influence for differences in subcutaneous adipose are likely minimal. Lastly, future work should consider implementing a cognitive assessment as part of the exclusion criteria for study entry.

In conclusion, fatigue resulted in a reduced BF coactivation amplitude for the DynCoact200ms (with age groups collapsed) and DynCoact10° variables. However, a close inspection of the data suggested a more severe reduction in the DynCoact10° variable in the young compared to the old men such that there may be a muting of the coactivation depression in old men, but this fatigue-induced antagonist coactivation depression in this variable needs further investigation to be more fully established. Any depression in BF coactivation immediately following fatigue was rapidly restored and remained restored for 30 min after fatigue. DynCoact10° may be a useful variable for investigating antagonist muscle coactivation, particularly at the knee joint. On the basis of these findings, future research may consider implementing a coactivation variable that solely captures the final phase of a dynamic movement for the antagonist to help elucidate the coactivation mechanisms involved in this phase of movement.

## Data Availability

The raw data supporting the conclusion of this article will be made available by the authors, without undue reservation.
