# Supplementary material for: Interaction between age and fatigue on antagonist muscle coactivation during an acute post-fatigue recovery phase
Source: Front Aging. 2022 Oct 3;3:1005080. doi: 10.3389/fragi.2022.1005080 (PMC9574075; doi:10.3389/fragi.2022.1005080)
Supplement: Supplementary file 1 [file Table1.docx]

Supplementary Material

| **Supplementary Table 1. Estimated marginal means (EMG RMS) for** **IsomCoact200ms, DynCoact200ms, and DynCoact10°through an acute recovery phase.** | | | |
| --- | --- | --- | --- |
|  | Mean ± SD | |  |
| **IsomCoact200ms** | |  |  |
| PRE | | 45.01 ± 32.07 |  |
| POST0 | | 40.93 ± 33.03 |  |
| POST7 | | 45.01 ± 32.31 |  |
| POST15 | | 43.80 ± 28.83 |  |
| POST30 | | 42.52 ± 30.82 |  |
| **DynCoact200ms** | |  |  |
| PRE | | 54.95 ± 37.95 |  |
| POST0 | | 46.52 ± 32.03 |  |
| POST7 | | 53.93 ± 36.01 |  |
| POST15 | | 50.16 ± 31.57 |  |
| POST30 | | 51.63 ± 34.57 |  |
| \| **DynCoact10º** \|  \|  \|  \| \| --- \| --- \| --- \| --- \| | |  |  |
| PRE | | 35.71 ± 25.88 |  |
| POST0 | | 27.56 ± 23.79 |  |
| POST7 | | 33.08 ± 26.11 |  |
| POST15 | | 34.30 ± 25.72 |  |
| POST30 | | 32.61 ± 23.12 |  |
| Abbreviations: RMS = root mean square; SD = standard deviation; IsomCoact200ms = Isometric BF coactivation RMS was calculated at 0-200 ms; DynCoact200ms = dynamic isokinetic 240°·s-1 BF coactivation RMS was calculated at 0-200 ms; DynCoact10º = BF coactivation for the final 10º of the leg extension on the isokinetic 240°·s-1 MVC; POST0 = 0 min into recovery; POST7 = 7 min into recovery; POST15 = 15 min into recovery; POST 30 = 30 min into recovery. | | | |
